# Experimental and Numerical Study on Shear Performance of Pitched Screws in Wood–Concrete Composite Beam with Wooden Partitions

**DOI:** 10.3390/ma16145098

**Published:** 2023-07-19

**Authors:** Shengnan Yuan, Hao Du, Zhixiang Sun, Xiamin Hu

**Affiliations:** 1 College of Civil Engineering, Nanjing Forestry University, Nanjing 210037, China; 2 College of Civil Engineering, Sanjiang University, Nanjing 210012, China

**Keywords:** wood–concrete composite beam, wooden partition, pitched screws, finite element analysis, shearing capacity

## Abstract

Wooden partitions are extensively used as formwork for pouring concrete in wood–concrete composite beams, especially in the restoring of wood structures. However, limited research has been conducted on the shear properties of pitched screw connectors in wood–concrete composite beams with wooden partitions. Therefore, this study investigated the shear performance of pitched screws in wood–concrete composite beams with wooden partitions through push-out tests and finite element analysis. The test results revealed that the failure mode of pitched screws was characterized by the pulling failure of the screws under tensile–shear action. The finite element analysis accurately predicted the failure mode, stress distribution, and load–slip behavior of pitched screws. Furthermore, the effects of the screw embedding angle, wooden partition thickness, concrete strength, and the length–diameter ratio of the screw were investigated through parametric analyses. It was found that when the screw diameter was 12 mm, the shearing capacity of the pitched screws with embedding angles of ±45°, ±60°, and ±75° decreased by 3.9%, 11.9%, and 26.9%, respectively, compared to the screws with an embedding angle of ±30°. The shearing capacity of pitched screws improved with the increase in the concrete strength and length–diameter ratio of the screw. However, the improvement in shearing capacity became less significant as the concrete strength and length–diameter ratio of the screw increased. Moreover, an increase in wooden partition thickness reduced the shearing capacity of pitched screws.

## 1. Introduction

The wood–concrete composite beam is composed of the wood beam and concrete flange, making full use of the mechanical properties of concrete under compression and wood under tension. The composite action is achieved through shear connections, which serve the purpose of transferring longitudinal shear forces at the interface and resisting the lift-up effect between the composite components. During the renovation and repair of traditional wood structures, the wooden partition is utilized as a construction platform for concrete pouring and retained as an integral part of the composite structure. The wood–concrete composite structures have gained significant traction in the construction industry and achieved applications in high-rise wooden buildings and bridge slab systems [1,2].

Currently, extensive experimental analyses and theoretical studies have been conducted on the shear behavior of screw connections [3,4,5,6,7,8,9,10,11]. Saulius et al. [3] conducted push-out tests on screw connections in wood–concrete composite beams, identifying three failure modes: non-hinged, single-hinged, and two-hinged. The calculation methods were proposed to determine the shearing capacity of screw connectors under each failure mode. Berardinucci et al. [6] conducted push-out tests on screw connectors in wood–concrete composite beams with and without wooden partitions. The results indicated that screw connectors in wood–concrete composite beams without partitions exhibited greater shear rigidity compared to those in wood–concrete composite beams with partitions. Symons et al. [7] compared screw connectors with different embedded angles and found that the pitched screw connectors had higher shear rigidity than the vertical embedded screws. Jorge et al. [9] investigated the interlayer influence on the shearing capacity of pitched screws in wood–lightweight-concrete composite beams. The results revealed that the screw connectors in composite beams with partitions had lower shearing capacity. Marchi et al. [11] conducted push-out tests on pitched screws to analyze the influence of screw diameter and concrete type on shearing capacity. The results indicated that the pitched screws exhibited higher shear rigidity in normal concrete compared to lightweight concrete.

In recent years, several finite element studies have been conducted on wood–concrete composite beams with screw connectors [12,13,14,15,16,17]. Lopes et al. [13] proposed a simplified method for determining the effective flexural stiffness of wood–concrete composite structures. The finite element modeling was utilized to optimize the strength prediction of the system, and it significantly influenced the evaluation of the optimal connection mode. Avez et al. [14] employed finite element methods to model joints with large clearances and pitched screws by simulating the interaction between screws and wood through the “cohesive surface.” The gap between the smooth shank of the screw and the outside diameter of the tapping screw was represented by filling it with a soft material, satisfying the requirements of the complex element modeling. Du et al. [15] performed numerical simulations on glulam–concrete composite beams with wooden partitions. The simulation results indicated that the ultimate strength and bending stiffness of composite beams decreased as the shear connection spacing increased. Oudjene et al. [16] developed a numerical method for studying the nonlinear behavior of wood–concrete composite beams with screw connections, utilizing one-dimensional beam element modeling for screws and three-dimensional solid element modeling for wood and concrete members in finite element analysis. Duong et al. [18] analyzed the bearing strength of screws in fiberglass-reinforced plastic structures. To address the complexity of the numerical simulation, the truss elements were employed to simulate the pull-out contact model.

Currently, there is limited research available on the shear properties of pitched screw connectors in wood–concrete composite beams with wooden partitions. Therefore, this paper aimed to address this gap by conducting an experimental study on pitched screw connectors in wood–concrete composite beams with wooden partitions. Additionally, the three-dimensional finite element analysis was performed using the ABAQUS 2020 finite element analysis software to analyze the behavior of the pitched screws. This study focused on investigating the influence of various factors, such as screw embedding angle, wooden partition thickness, concrete strength, and screw length–diameter ratio on the shearing capacity of the pitched screws.

## 2. Push-Out Test Study

### 2.1. Test Specimens

**Figure 1:**
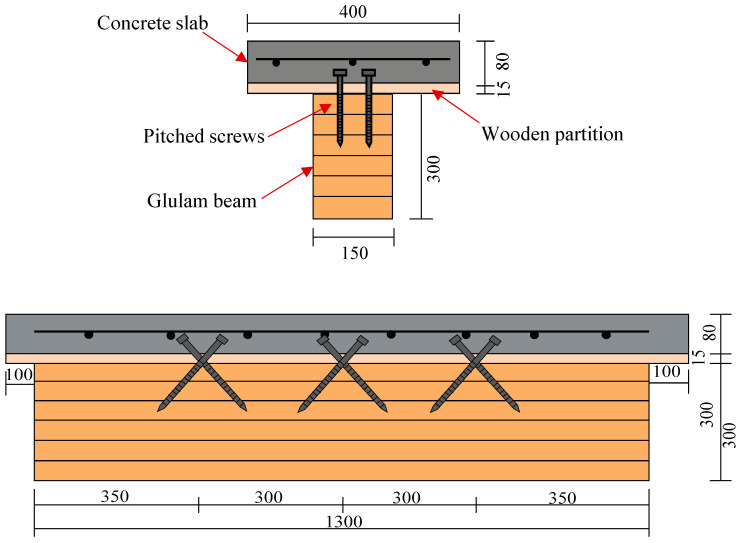
Dimension of push-out specimen.

**Figure 2:**
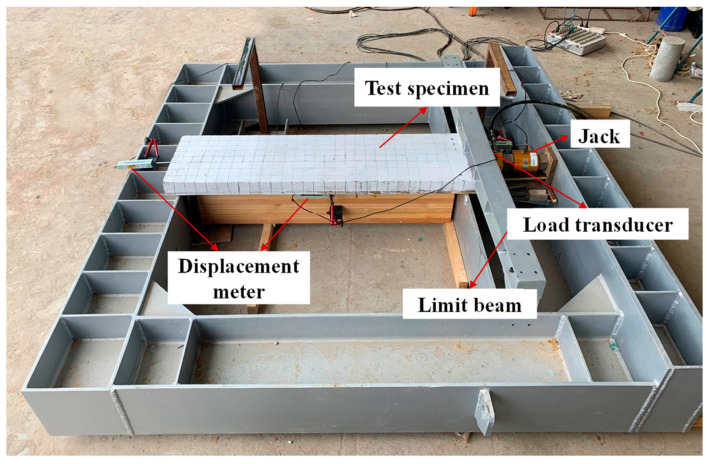
Loading equipment of push-out tests.

Two glulam–concrete specimens (GCS-1 and GCS-2) were constructed and tested under static load. The detailed parameters of the test specimens are depicted in Figure 1. Each glulam–concrete composite specimen consisted of one 150 × 300 ×1300 mm glulam member and one 80 × 400 × 1500 mm concrete slab. Wooden partitions with a thickness of 15 mm and a width of 400 mm were installed on top of the wood member to serve as the formwork for pouring the concrete slab. Three pairs of pitched screws were installed at the wood–concrete interface with an inclined angle of 45°. The screws had a diameter of 12 mm, and their lengths embedded into the wood beam and concrete slab were 100 mm and 70 mm, respectively. The wood–concrete composite beam was positioned within a self-balancing frame. A load sensor and a jack were arranged at the end of the composite beam to apply the load on the concrete slab. Displacement meters were strategically placed at the end and middle of the test specimens to monitor the interface displacement between the concrete slab and wood beam during the loading. Figure 2 illustrates the specific loading device employed in this study. The loading scheme of the push-out tests was determined in accordance with EN 26891 [19].

The wood used in the test specimens had an average moisture content of 11.3% and an average air-dry density of 0.58 g/cm^3^. The compressive elastic modulus parallel to the wood grain was determined to be 12,570 N/mm^2^. Furthermore, the average compressive strength parallel to the wood grain was found to be 57.6 N/mm^2^, and the shearing capacity parallel to the wood grain was measured as 4.3 N/mm^2^. The cylinder compression strength of the concrete was determined to be 26.2 N/mm^2^. The screw fasteners had a tensile strength of 461.6 N/mm^2^.

### 2.2. Push-Out Test Result

**Figure 3:**
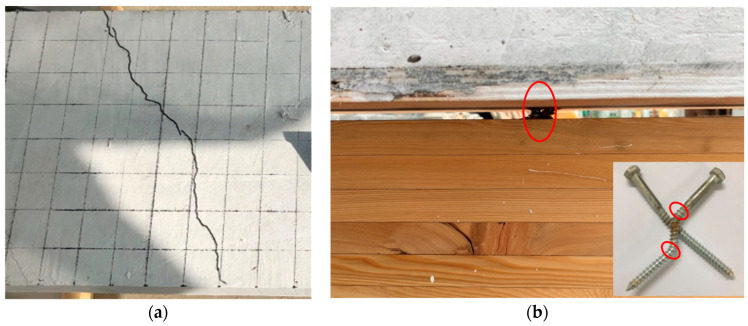
Push-out test phenomenon. (**a**) Cracks of concrete slab; (**b**) Pulling out failure of screw fasteners.

During the initial loading stage, the wood–concrete composite beam exhibited good mechanical performance, with minimal relative slip and lifting displacement between the concrete slab and wood beam. However, as the applied load increased, various cracks and damages were observed. At a load of 109 kN, the cracks were observed at the upper part of the concrete slab, as shown in Figure 3a. Additionally, a distinct tearing sound was audible, indicating significant damage to the wood beam and screw connectors. Subsequently, when the load reached 136 kN, the concrete slab slid in the direction of the applied force, leading to the extraction of the screw fastener from the wood beam (Figure 3b). The observed failure mode of the push-out specimens was the pulling out failure of the screw fasteners, resulting from the combination of tensile and shear forces.

**Figure 4:**
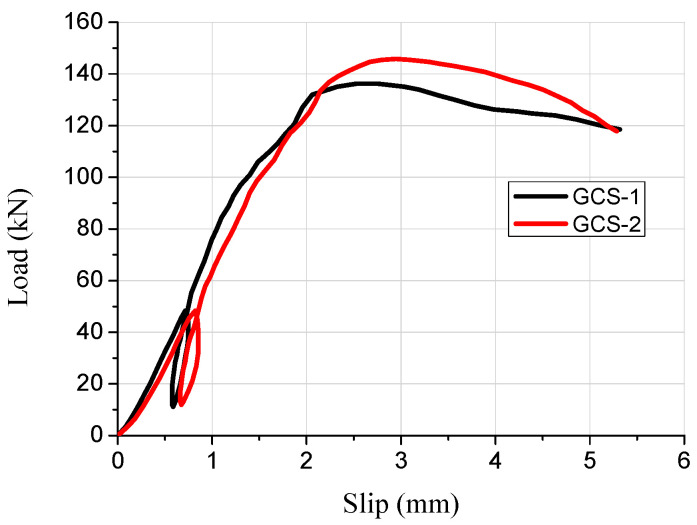
Load–slip curves of pitched screws.

During the push-out tests, the load–slip curves of the pitched screws in the wood–concrete composite beam with wooden partitions were obtained through continuous monitoring, as shown in Figure 4. Initially, the slip curves exhibited linear growth as the load was applied. However, as the load increased further, the slip curves displayed nonlinear growth. This demonstrated that the shear rigidity of the pitched screws continuously reduced with the increase in the load. The reduction in shear rigidity could be attributed to the significant bending deformations experienced by the screw connections under shear forces. As the load reached the ultimate value, the load began to decrease slowly while the slip continued to increase. This behavior indicated the pitched screw connectors had progressive failure characteristics. Overall, the pitched screws exhibited good ductility. Furthermore, the average shearing capacity of one pair of pitched screws was determined to be 46 kN. The test results provided valuable insights into the shear performance of the pitched screws in the wood–concrete composite beam with wooden partitions.

## 3. Finite Element Model

### 3.1. Constitutive Relations of Materials

To investigate the shear properties of pitched screw connectors in wood–concrete composite beams with wooden partitions, a three-dimensional finite element model was developed using the ABAQUS 2020 software. According to the research results in the literature [20], a plastic damage model was adopted for the concrete material. The assumption was made that the tensile strength of the concrete exhibited a linear relationship with its compressive strength. The elastic modulus and cylinder compression strength of concrete in the finite model were 32.5 GPa and 26.2 MPa, respectively. The peak strain of the concrete was taken as 0.0018 in this model. The stress–strain relationship equation under uniaxial compression is shown as follows:

(1) $$\left\{\begin{array}{c} y=ax+\left(3-2a\right)x^{2}+\left(a-2\right)x^{3}\\ y=\frac{x}{b{\left(x-1\right)}^{2}+x} \end{array}\right.\begin{array}{c} x\leq 1\\ x>1 \end{array}$$

where $x=\varepsilon_{c}/\varepsilon_{0}$; $y=\sigma_{c}/f_{c}$; $\varepsilon_{0}$ = peak strain; $f_{c}$ = concrete strength corresponding to the peak strain; *a* and *b* = calculation coefficient, *a* is taken as 1.7, and *b* is taken as 2.0 in this model.

The wood material in this model was simplified as an orthotropic material. The relationship between the elastic modulus and shear modulus of wood was determined based on the guidelines provided by EN 338 [21]. The orthotropic yield standard was employed to model the behavior of wood [22]. The compressive elastic modulus parallel to the wood grain in the finite model was taken as 12570 MPa. The compressive elastic modulus perpendicular to the grain was 420 MPa, and the shear modulus was 786 MPa. For the steel material, the constitutive relation followed an elastic-strengthening model, which was represented by two broken lines [23]. This model accounts for the nonlinear behavior and strengthening effect observed in steel materials under load. The elastic modulus and yield strength of the steel in the finite model were 210 GPa and 380 MPa, respectively.

### 3.2. Interface Contact and Boundary Conditions

In the finite element simulation, the concrete slabs, wood beams, screw connectors, and wooden partitions were modeled using three-dimensional solid reduced integral elements known as C3D8R elements. These elements were hexahedral in type and linear in geometric order. The surface contact setup in ABAQUS was utilized to simulate the interaction between different components. The friction model “penalty” was used in the tangential direction to account for the frictional behavior, while hard contact was set in the normal direction to prevent interpenetration. The type of applied load was concentrated force, with the direction perpendicular to the side of the concrete slab. To facilitate the application of loads, the reference points were established in a coupling manner. The coupling type was selected as continuous distribution, ensuring the load transfer between the components. Based on the boundary conditions of the push-out test, the translational degrees of freedom and rotational degrees of freedom along the X, Y, and Z directions were constrained at one end of the glued wood beam. Similarly, the translational degrees of freedom of the concrete slab were restricted at the other end, ensuring proper fixation of the specimen during the simulation. The analysis step type for the solution procedure is static and universal, considering the geometric nonlinearity of the material. The Newton–Raphson iterative method was used in the nonlinear solution procedure.

## 4. Finite Element Results and Discussions

### 4.1. Finite Element Model Verification

**Figure 5:**
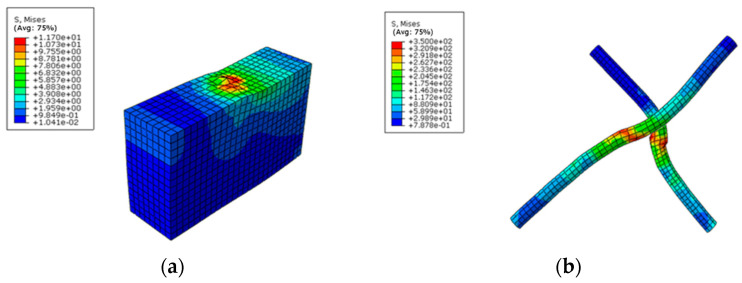
Deformation and stress program of screw fasteners. (**a**) Wood beam; (**b**) Pitched screws.

**Figure 6:**
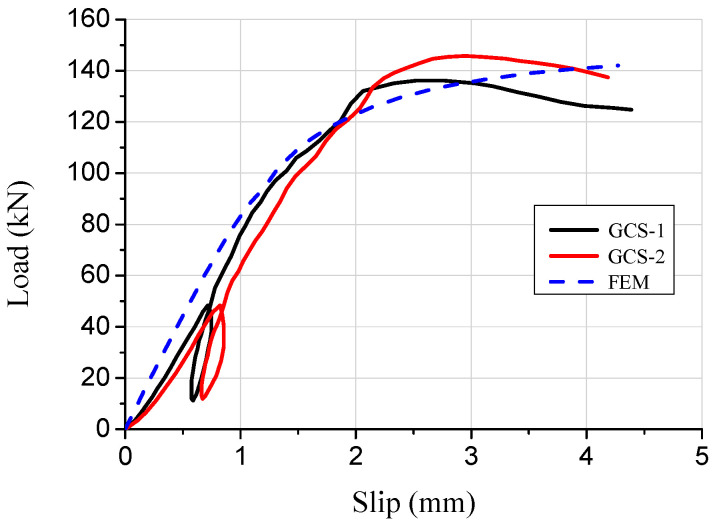
Comparison of load–slip curves between finite element simulation and test.

Based on the numerical results, the stress–strain behavior of the concrete slab, wooden partition, wood beam, and screw connectors in the specimen was analyzed. The failure mode observed in the composite specimen under the limit state was the pulling out of the pitched screw connectors under tensile shear action, with the concentration of stresses mainly occurring at the joint of the pitched screws (Figure 5a). The screw fasteners under compression shear forces exhibited bending failure with double hinges, which was consistent with the observed destruction mechanism in the push-out test (Figure 5b). The results indicated that the finite element simulation accurately predicted the failure mode and stress distribution of the pitched screws. Figure 6 presents a comparison of the load–slip curves obtained from the finite element simulation and the push-out test. The average discrepancy between the finite element results and the test results was 15.4%. It was observed that the presented finite element model could adequately predict the load–slip response of pitched screws, especially in the elastic range. The discrepancy between the finite element results and the test results increased in the plastic range, which could be attributed to the simplified constitutive law of wood used in the finite element model. The shearing capacity predicted by the finite element simulation also matched well with the experimental results. During the initial loading stage, the slip curve exhibited linear growth with increasing load, and the relative slip was minimal due to the adhesion and friction between the wood beams and concrete. As the applied load increased, the slip curve showed nonlinear growth, indicating a continuous deterioration of the shear rigidity of the screw connectors.

### 4.2. Parametric Study

#### 4.2.1. Effect of the Screw Insertion Angle

**Figure 7:**
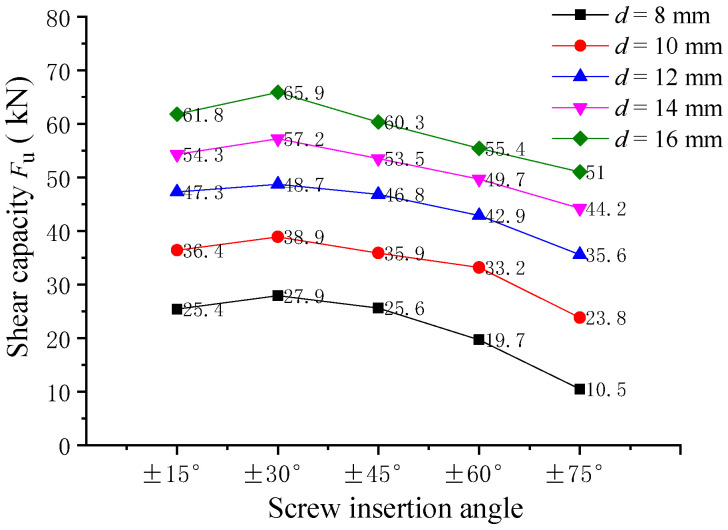
Effect of the screw insertion angle.

The influence of the screw embedding angle on the shearing capacity of pitched screws was investigated through finite element analysis. The analysis considered screw connectors with embedding angles of ±15°, ±30°, ±45°, ±60°, and ±75°, and the simulation results are shown in Figure 7. It was observed that the trend of shearing capacity variation for the pitched crossing screw connectors with different screw embedding angles was similar. The shearing capacity of the screws with an embedding angle of ±30° was the highest. Specifically, when the screw diameter was 12 mm, the shearing capacity of the pitched screws with embedding angles of ±45°, ±60°, and ±75° decreased by 3.9%, 11.9%, and 26.9%, respectively, compared to the screws with an embedding angle of ±30°. It indicated that as the screw embedding angle increased from ±30° to ±75°, the shearing capacity of the pitched screw connections decreased. The main reason for this reduction in shearing capacity was the reduction of the rope effects provided by the screws. In summary, the finite element analysis revealed that the shearing capacity of pitched screw connections was significantly influenced by the screw embedding angle, with an embedding angle of ±30° providing the highest shearing capacity. As the embedding angle deviated from ±30°, the shearing capacity gradually decreased due to the reduced rope effects of the pitched screw fasteners.

#### 4.2.2. Effect of the Wood Partition Thickness

**Figure 8:**
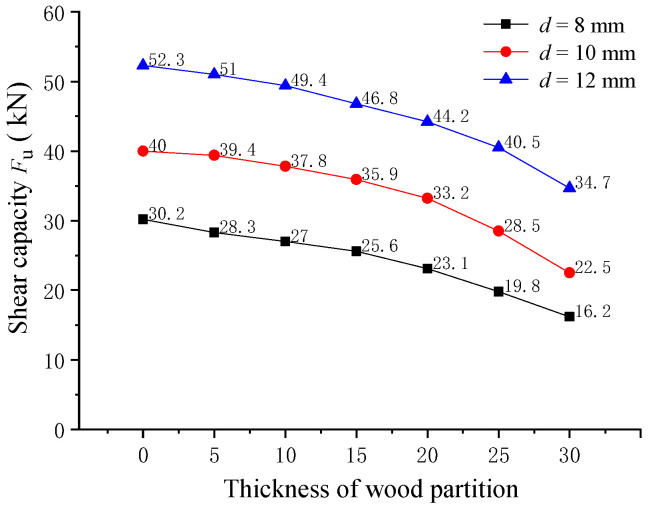
Effect of the wooden partition thickness.

The influence of wooden partition thickness on the shearing capacity of pitched screws was examined through finite element analysis. The composite beam specimens with wooden partition thicknesses ranging from 0 mm to 30 mm were considered in the analysis. The finite element results of the shearing capacity of pitched screws are shown in Figure 8. It was observed that the changing trend of shearing capacity for pitched screws with different screw diameters was similar. As the wooden partition thickness increased, the shearing capacity of the pitched screws gradually decreased. Specifically, when the wooden partition thickness was 5, 10, 15, 20, 25, and 30 mm, the shearing capacity of the pitched screws decreased by 2.5%, 5.6%, 10.5%, 15.5%, 22.6%, and 33.7%, respectively, compared to the screw connectors in wood–concrete composite beams without wooden partitions. These findings indicated that the presence of a wooden partition in the composite beam had a negative effect on the shearing capacity of the pitched screws. As the wooden partition thickness increased, it separated the wood beam and concrete slab, reducing the effectiveness of the screw connectors in transferring shear forces. Consequently, the shearing capacity of the pitched screws decreased as the wooden partition thickness increased. Overall, the finite element analysis provided insights into the influence of wooden partition thickness on the shearing capacity of pitched screws, highlighting the importance of considering the presence and thickness of wooden partitions when designing wood–concrete composite structures with pitched screw connectors.

#### 4.2.3. Effect of the Concrete Strength

**Figure 9:**
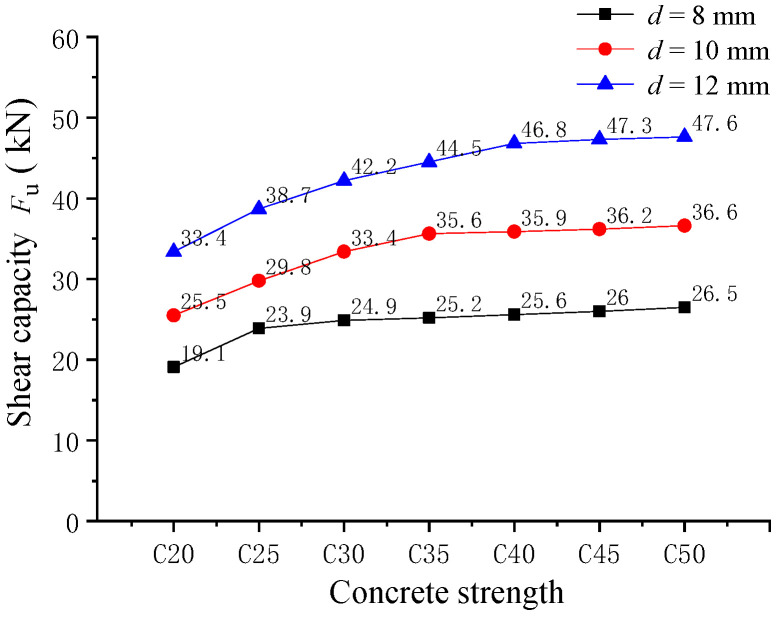
Effect of the concrete strength.

The effect of concrete strength on the shearing capacity of pitched screws was investigated through finite element analysis. The pitched screw connectors were analyzed, with concrete strengths ranging from C20 to C50. The analysis results of the shearing capacity are presented in Figure 9. It was observed that the variation trend of shearing capacity for three different screw diameters was generally similar. When the screw diameter was 12 mm, compared to the screw connectors in composite beams with the concrete strength of C20, the shearing capacity of the pitched screw connectors with concrete strengths of C25, C30, C35, C40, C45, and C50 increased by 15.8%, 26.3%, 33.2%, 40.1%, 41.6%, and 42.5%, respectively. With the increase in concrete strength, the shearing capacity of the pitched screw connectors gradually increased and tended to level off. This was because the higher concrete strength provides better load-carrying capacity, allowing for increased shear transfer between the wood beam and concrete slab through the pitched screws. However, it should be noted that the influence of concrete strength on the shear properties of pitched screws gradually diminished, as the elastic modulus of concrete was much larger than that of wood. Therefore, the increase in concrete strength had a diminishing effect on the shear properties of the pitched screw connectors. These findings suggested that higher concrete strength could enhance the shearing capacity of pitched screw connectors in wood–concrete composite beams. However, the magnitude of this enhancement diminished as the concrete strength increased. Therefore, when selecting and designing pitched screw connectors for wood–concrete composite structures, the influence of concrete strength should be considered to ensure optimal shear performance.

#### 4.2.4. Effect of the Length–Diameter Ratio of Screw

**Figure 10:**
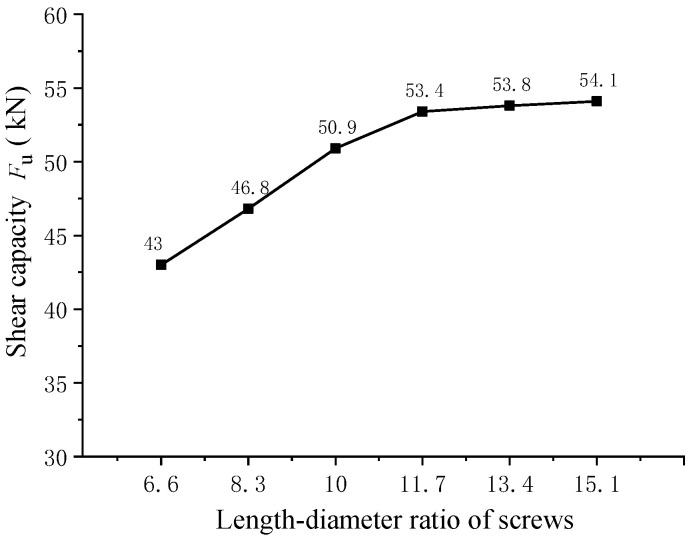
Effect of the length–diameter ratio of screws.

To analyze the influence of the screw length–diameter ratio on the shear resistance of pitched screws, a finite element analysis was conducted on pitched screw connectors with different length–diameter ratios (Figure 10). It was observed that the shearing capacity of pitched screws increased as the length–diameter ratio increased. Compared to the screws with a length–diameter ratio of 6.6, the shearing capacity of pitched screws with length–diameter ratios of 8.3, 10, 11.7, 13.4, and 15.1 was improved by 8.8%, 18.4%, 24.2%, 25.1%, and 25.8%, respectively. The improvement in shearing capacity could be attributed to the increased withdrawal strength of the screw fasteners with longer length–diameter ratios. A higher length–diameter ratio allows for a greater embedment depth of the screws, resulting in improved resistance to pull-out forces. Therefore, the increasing in the length–diameter ratio enhanced the shearing capacity of pitched screws. However, it should be noted that the rate of increase in shearing capacity gradually decreased as the length–diameter ratio increased. When the length–diameter ratio of the screw reached 11.7, the shearing capacity improvement became more moderate. In conclusion, the results indicated that the shearing capacity of pitched screws was improved by increasing the length–diameter ratio due to the enhanced withdrawal strength of the screw fasteners. However, the rate of improvement diminished as the length–diameter ratio increased. The optimal length–diameter ratio of the screw for maximizing shearing capacity was found to be approximately 11.7. This information can be valuable for designing pitched screw connectors in wood–concrete composite structures, allowing for better optimization of the screw geometry to achieve the desired shear performance.

## 5. Conclusions

The experimental and numerical investigations conducted in this study provided valuable insights into the shear performance of pitched screws in wood–concrete composite beams with wooden partitions. The following key findings were observed:

(1) The failure mode of pitched screws was characterized as the pulling failure of the screw fasteners under tensile shear action. The load–slip curve exhibited linear growth initially, with a relatively small slip. As the applied load increased, the curve showed nonlinear growth, indicating a reduction in the shear rigidity of the screw connectors. At the ultimate load, the load began to decrease gradually with increasing slip, demonstrating progressive failure and good ductility of the pitched screw connectors. The finite element analysis accurately predicted the failure mode, stress distribution, and load–slip behavior of pitched screws.
(2) When the screw diameter was 12 mm, the shearing capacity of the pitched screws with embedding angles of ±45°, ±60°, and ±75° decreased by 3.9%, 11.9%, and 26.9%, respectively, compared to the screws with an embedding angle of ±30°. The shearing capacity of pitched screw connections decreased as the screw embedding angle increased beyond 30°. This reduction could be attributed to the diminishing rope effects of the screw fasteners. Additionally, an increase in wooden partition thickness led to a gradual decrease in the shearing capacity of the pitched screw connectors.
(3) Compared to the screws with a length–diameter ratio of 6.6, the shearing capacity of pitched screws with length–diameter ratios of 8.3, 10, 11.7, 13.4, and 15.1 was improved by 8.8%, 18.4%, 24.2%, 25.1%, and 25.8%, respectively. The shearing capacity of pitched screws improved with an increase in the length–diameter ratio due to the enhanced withdrawal strength of the screw fasteners. However, the rate of improvement became less significant as the length–diameter ratio increased. The optimal length–diameter ratio was found to be approximately 11.7.
(4) The shearing capacity of pitched screw connectors increased with an increase in concrete strength. However, the rate of increase became more gradual as the concrete strength increased. This effect could be attributed to the relatively large elastic modulus of concrete compared to wood, causing a diminishing influence of concrete strength on the shear properties of the pitched screws.

The present study focused on analyzing the shear performance of pitched screws in wood–concrete composite beams with wooden partitions. The results can inform the design and optimization of pitched screw connectors in wood–concrete composite structures, enabling improved shear resistance and overall structural performance. A subsequent paper will investigate the shear performance of notched connectors and steel plate connectors in wood–concrete composite beams with wooden partitions. Furthermore, it is recommended that future research should also explore the shear performance of pitched screws in wood–concrete composite beams under fire conditions.

## Data Availability

Not applicable.
